# Nitrogen Use Efficiency in Parent vs. Hybrid Canola under Varying Nitrogen Availabilities

**DOI:** 10.3390/plants10112364

**Published:** 2021-11-02

**Authors:** Shanay T. Williams, Sally Vail, Melissa M. Arcand

**Affiliations:** 1 Department of Soil Science, University of Saskatchewan, Saskatoon, SK S7N 5A8, Canada; 2 Saskatoon Research Centre, Agriculture and Agri-Food Canada, Saskatoon, SK S7N 0X2, Canada; sally.vail@canada.ca

**Keywords:** canola, nitrogen use efficiency, N availability, N mineralization, potential ammonium oxidation rates, fertilizer rates, phenological stage

## Abstract

Improving nitrogen use efficiency (NUE) is essential for sustainable agriculture, especially in high-N-demanding crops such as canola (*Brassica napus*). While advancements in above-ground agronomic practices have improved NUE, research on soil and below-ground processes are limited. Plant NUE—and its components, N uptake efficiency (NUpE), and N utilization efficiency (NUtE)—can be further improved by exploring crop variety and soil N cycling. Canola parental genotypes (NAM-0 and NAM-17) and hybrids (H151857 and H151816) were grown on a dark brown chernozem in Saskatchewan, Canada. Soil and plant samples were collected at the 5–6 leaf stage and flowering, and seeds were collected at harvest maturity. Soil N cycling varied with phenotypic stage, with higher potential ammonium oxidation rates at the 5–6 leaf stage and higher urease activity at flowering. Seed N uptake was higher under higher urea-N rates, while the converse was true for NUE metrics. Hybrids had higher yield, seed N uptake, NUtE, and NUE, with higher NUE potentially owing to higher NUtE at flowering, which led to higher yield and seed N allocation. Soil N cycling and soil N concentrations correlated for improved canola NUE, revealing below-ground breeding targets. Future studies should consider multiple root characteristics, including rhizosphere microbial N cycling, root exudates, and root system architecture, to determine the below-ground dynamics of plant NUE.

## 1. Introduction

Canola (*Brassica napus*) is an important oilseed crop grown globally, with Canada producing over 20.7 and 19.6 million metric tons of canola in 2018 and 2019, respectively [1], making Canada one of the largest canola producers globally. Canola is used in human consumption, animal feed, and feedstock for biofuels, requiring relatively large inputs of N to produce high yields [2,3,4,5,6,7,8,9]. However, there are negative environmental concerns, with excessive N fertilizer use affecting the soil and waterways through nitrate leaching and runoff [10]. The future of sustainable agriculture requires increased and stable crop yields, with decreasing amounts of N fertilization [11,12,13] to protect ecosystem health while feeding the growing population. Thus, researchers must find solutions to maximize agronomic and economic competitiveness while reducing associated negative environmental impacts [14]. Agronomic and economic competitiveness is achievable by improving and enhancing crop N use efficiency (NUE) [11].

Improvements in NUE and seed yield traits in hybrid varieties are crucial outcomes in the development of canola [15,16,17,18]. The selection of enhanced NUE traits in hybrid canola might have occurred passively; for example, winter canola hybrids outcompeted older varieties concerning NUE traits regardless of N fertilizer rates [18]. High NUE in hybrid canola also correlates to specific traits, including increased plant biomass at flowering and increased seed yield [17]. Higher NUE in spring hybrid canola may also be associated with below-ground interactions, including better scavenging of soil and fertilizer N [15].

Crop NUE consists of two components: N uptake efficiency (NUpE) and N utilization efficiency (NUtE) [19]. NUpE represents the plant’s ability to absorb N from the soil (whether derived from native soil N or fertilizer N), while NUtE represents the plant’s ability to partition absorbed N to specific plant organs [19]. However, of the two components, NUpE is more directly influenced by the soil and root–soil interactions. Thus, plants affect NUE by regulating N uptake rates, modifying biomass allocation, and adapting root system architecture to improve N capture [20,21,22]. Genotypic variability in NUpE and NUtE have been observed in numerous agriculturally relevant crops, including wheat (*Triticum aestivum*) [23,24], maize (*Zea mays*) [25], rice (*Oryza sativa*) [26,27], barley (*Hordeum vulgare*) [28,29], and canola (*Brassica napus*) [17,18].

Crop breeding has focused efforts on developing crop varieties capable of scavenging plant-available soil N [30]. Plant inherited traits are involved in soil N processes across different crop species [23,31,32,33]. Pathan et al. (2015) reported that a maize variety with higher NUE could induce faster soil inorganic N depletion in the rhizosphere than a variety that had lower NUE [33]. The high NUE maize variety also had a distinct microbial community structure and soil extracellular enzyme activity, potentially increasing inorganic N supply than the low NUE variety [33]. The synchronicity between below-ground processes and plant N status, growth, and productivity is complex due to the dynamic soil processes at play. Plant roots stimulate and select distinct microbial communities, which access plant carbon and hydrolyze organic N to inorganic N forms. A better understanding of the below-ground synchronicity between plant N demand and soil N cycling can improve crop NUE further. For example, crop breeding has altered soil N acquisition, with newer maize varieties exhibiting increased soil N cycling processes and crop N uptake [34]. Further, higher mineralization and nitrification rates enhanced NUE under high NUE maize than the low NUE maize varieties [35]. To our knowledge, no such data exist on soil N cycling, plant nitrogen uptake, and NUE components for spring canola; however, this could inform canola breeding programs’ efforts in selecting varieties that can exploit naturally occurring processes to improve NUE.

Our objective for this study was to evaluate how (1) soil N cycling processes affecting plant-available N, (2) soil inorganic N concentrations, and (3) canola NUE (including NUpE and NUtE) differed between canola varieties (hybrids vs. parent genotypes) grown under varying urea-N fertilizer rates. Two important reactions associated with the concentrations of soil NH_4_^+^ and NO_3_^−^ include the hydrolysis of urea—a predominant source of N fertilizer in agricultural soils—to NH_4_^+^ by urease enzyme [36], and the oxidation of NH_4_^+^ to nitrite (NO_2_^−^) during the first step of nitrification by ammonia monooxygenase [37]. Plants mainly absorb NH_4_^+^-N and NO_3_^−^-N [38], though canola preferentially absorbs NO_3_^−^-N [39]. Urease produces NH_4_^+^-N, and urease activity is responsive to urea-N fertilizer [40], while nitrification results in the formation of NO_3_^−^-N, and potential ammonium oxidation is an indicator of nitrification [41,42]. Thus, the soil N cycling processes we focused on were urease activity and potential ammonium oxidation because they affect plant-available N. We examined whether soil N cycling, NUpE, and NUtE varied with phenological stage within the growing season (5–6 leaves and flowering) and the resulting NUE at crop harvest. We hypothesized that canola varieties with higher plant NUE (and the two NUE components: NUpE and NUtE) would be associated with increased soil N cycling and passive plant trait yield components like higher thousand-seed weight, earlier start to flowering, and longer duration of flowering. Passive plant traits can affect canola yield and NUE for different canola varieties [17,18]; these traits, like earlier flowering and longer flowering duration, were also hypothesized to positively influence hybrid yields and NUE in our study.

## 2. Results

### 2.1. Category A Variables: Within Growing Season Soil N Cycling and Crop NUE Components

#### 2.1.1. Soil Inorganic N Concentrations

**Figure 1:**
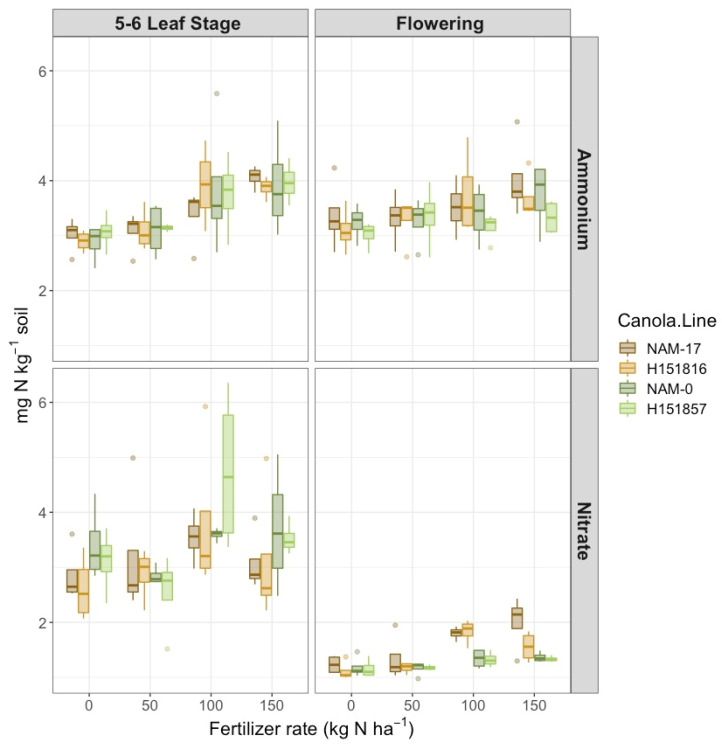
Soil ammonium-N concentration (mg NH_4_^+^-N kg^−1^ soil) and soil nitrate concentration (mg NO_3_^−^-N kg^−1^ soil) over four diverse canola varieties and four varying urea-N treatment rates at two phenological growth stages (5–6 leaf stage and flowering, *n* = 4).

There was a two-way interaction between fertilizer N rates and phenological stage on soil NH_4_^+^-N concentration (Table 1), where NH_4_^+^-N concentration was highest under the 150 kg ha^−1^ N rate at both phenological stages and lowest under the 0 kg ha^−1^ N rate at the 5–6 leaf stage (Figure 1). Canola varieties did not affect soil NH_4_^+^-N nor soil NO_3_^−^-N concentrations. However, there was a two-way interaction between canola variety and phenological stage on soil NO_3_^−^-N concentration (Table 1). Soil NO_3_^−^-N concentrations were highest and highly variable at the 5–6 leaf stage, but unlike soil NH_4_^+^-N, soil NO_3_^−^-N was less variable at flowering (Figure 1). At flowering, soil NO_3_^−^-N was lowest under the experimental hybrid H151857 and its parental genotype NAM-0, and was highest under NAM-17; soil NO_3_^−^-N was most similar between hybrid–parent pairs (Figure 1). Soil NO_3_^−^-N concentrations increased with increasing urea-N fertilizer rates at both phenological stages (Figure 1).

#### 2.1.2. Urease Activity and Potential Ammonium Oxidation Rates

**Figure 2:**
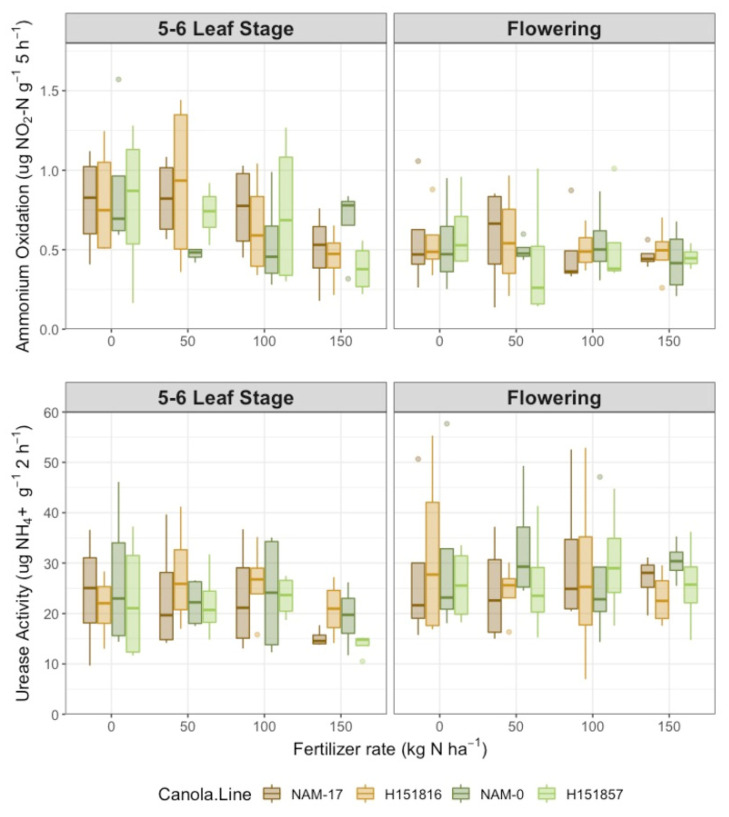
Potential ammonium oxidase rate (µg NO_2_-N g^−1^ 5 h^−1^) and urease activity (µg NH_4_^+^ g^−1^ 2 h^−1^) over four diverse canola varieties and four varying urea-N treatment rates at two phenological growth stages (5–6 leaf stage and flowering, *n* = 4).

Urease activity varied with phenological stage (Table 1); it was 20.0% higher at flowering than at the 5–6 leaf stage (Figure 2). However, urease activity was not affected by either canola variety or fertilizer N rate. Similarly, potential ammonium oxidation rate was not affected by canola variety or N rate but changed with phenological stage (Table 1). Inverse to urease activity, potential ammonium oxidation rates were 24.9% higher at the 5–6 leaf stage than at flowering (Figure 2). Soil pH varied with phenological stage (Table 1), with higher pH at flowering versus the 5–6 leaf stage.

#### 2.1.3. Canola Nitrogen Use Efficiency Components

Above-ground plant biomass varied with two-way interactions between (1) urea-N fertilizer rates and canola varieties and (2) canola varieties and phenological stage (Table 1). Plant biomass was highest under H151816 at 100 kg ha^−1^ N, and lowest under NAM-17 at 50 kg ha^−1^ N; parental genotypes had the lowest plant biomass at the 5–6 leaf stage but the highest biomass at flowering (Figure S1). Plant N uptake also varied with two-way interactions between (1) fertilizer N rates and canola varieties and (2) canola varieties and phenological stage (Table 1). Plant N uptake, like above-ground biomass, was highest under H151816 at the 100 kg ha^−1^ N rate and lowest under NAM-17 at the 50 kg ha^−1^ N rate (Figure S2). At the 5–6 leaf stage, H151857 and its corresponding parental genotype, NAM-0, had the highest N uptake; however, at flowering, both hybrids had lower N uptake than the parental genotypes (Figure S2).

**Figure 3:**
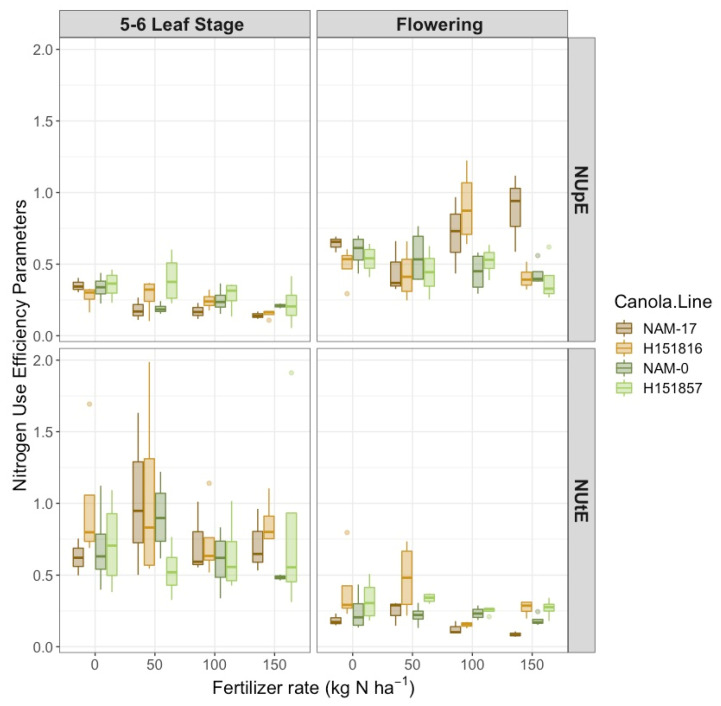
Canola (**top**) N uptake efficiency and (**bottom**) N utilization efficiency over four diverse canola varieties and four varying N treatment rates at two phenological growth stages (5–6 leaf stage and flowering, *n* = 4).

Nitrogen uptake efficiency (NUpE) was affected by a two-way interaction between canola varieties and phenological stage (Table 1). Hybrid H151816 and its corresponding parental genotype NAM-17 had the highest NUpE at flowering, while H151857 and its corresponding parental genotype, NAM-0, had the highest NUpE at the 5–6 leaf stage (Figure 3). There was also a two-way interaction between urea-N fertilizer rates and phenological stage on NUpE (Table 1). Plant NUpE decreased with increasing urea-N rates at the 5–6 leaf stage; however, NUpE was highest at the 0 and 100 kg ha^−1^ N rates at flowering (Figure 3).

Like NUpE, plant NUtE was affected by a two-way interaction between canola varieties and phenological stage (Table 1). At the 5–6 leaf stage, H151816 and its corresponding parental genotype, NAM-17, had the highest NUtE compared to H151857 and its corresponding parental genotype, NAM-0. However, both hybrids tended to have higher NUtE than the parental genotypes at flowering (Figure 3). Fertilizer N rates tended to affect plant NUtE (Table 1). Like NUpE, NUtE decreased with increasing N rates, with the 0 and 50 kg ha^−1^ N rates generally having the highest NUtE. Experimental hybrid H151816 had the highest NUtE at 50 kg ha^−1^ N, while its parental genotype, NAM-17, had the lowest NUtE at 150 kg ha^−1^ N.

### 2.2. Category B Variables: Within Season Crop Growth and Harvest Metrics

#### 2.2.1. Flowering Time

The start of flowering among canola varieties depended on urea-N fertilizer rate (Table 2). Genotype NAM-17 and its associated hybrid, H151816, tended toward a later onset flowering at higher N rates, while NAM-0 and its associated hybrid, H151857, tended towards an earlier onset flowering at lower N rates. The end of flowering was affected by canola variety and N fertilizer rate, but there was no interaction between these two factors (Table 2). Parent genotype NAM-17 finished flowering later than all other varieties. Flowering ended under the 0 kg ha^−1^ N controls before any of the urea-N fertilized treatments. The flowering duration was affected by canola variety and urea-N fertilizer rate, but there was no interaction between the two factors (Table 2). Both NAM-17 and its associated hybrid, H151816, had the shortest flowering duration of all genotypes, and flowering duration was shorter under the 0 kg N ha^−1^ plots.

#### 2.2.2. Yield, Seed Nitrogen Uptake, and Nitrogen Use Efficiency

**Figure 4:**
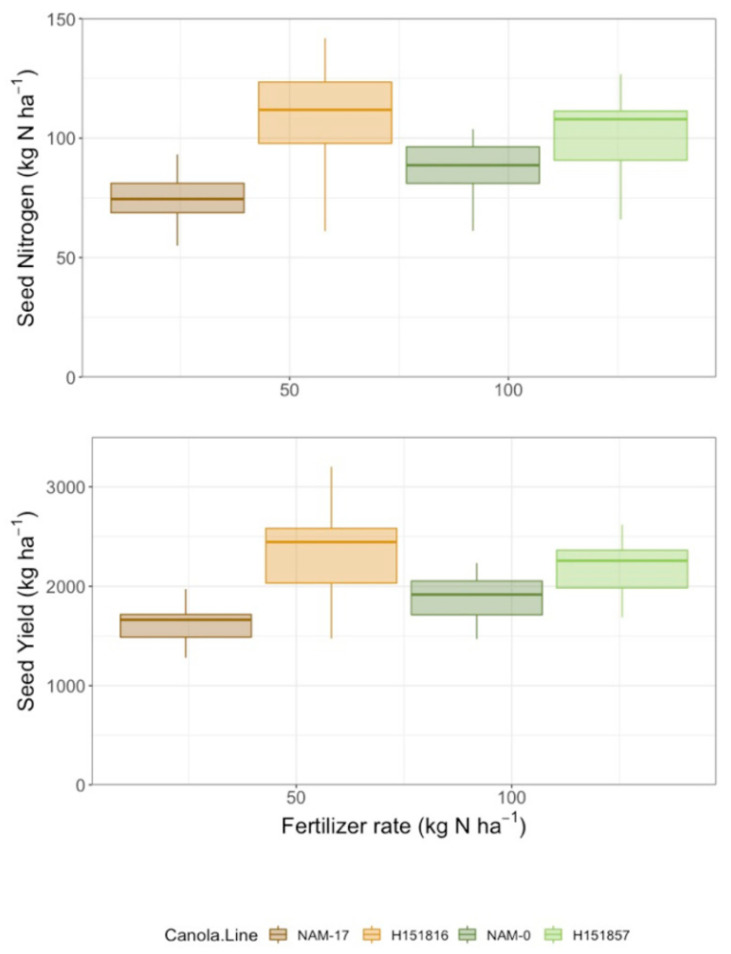
Yield (kg ha^−1^) and seed N uptake (kg ha^−1^) over four diverse canola varieties and four varying N treatment rates (*n* = 4).

**Figure 5:**
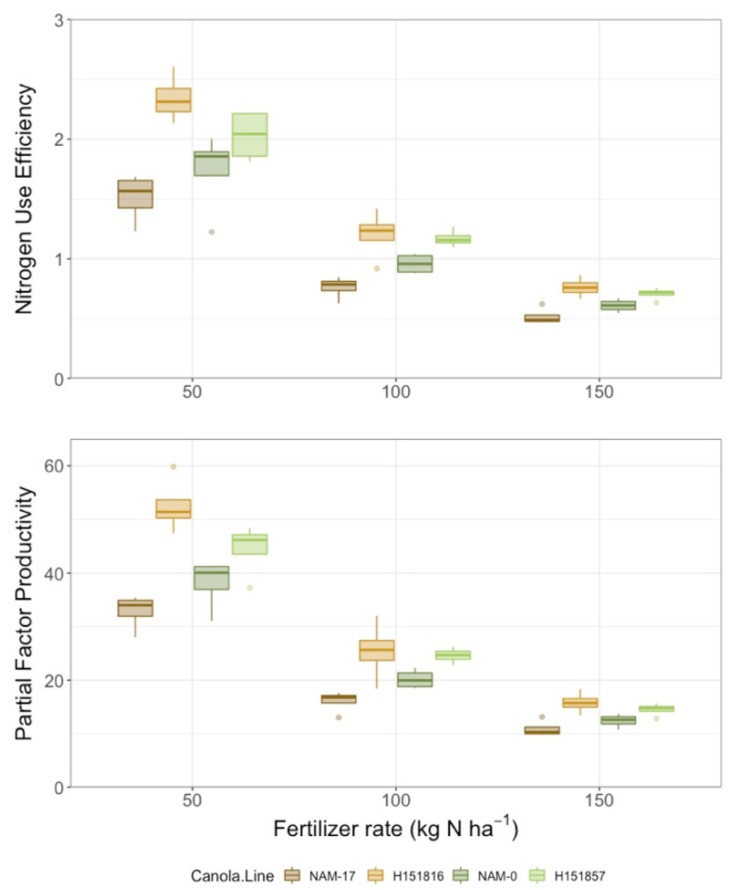
Nitrogen use efficiency (NUE) and partial factor productivity over four diverse canola varieties and three varying N treatment rates (*n* = 4).

Yield differed among canola varieties and urea-N fertilizer rates, but there was no interaction between these two factors (Table 2). Seed yield was lowest under both parental genotypes and highest under both hybrids; urea-N fertilizer treatment increased seed yield at all application rates relative to the 0 kg ha^−1^ N control (Figure 4). Similarly, seed N uptake differed among canola varieties and urea-N fertilizer rates, but there was no interaction between these two factors (Table 2). Like yield, seed N uptake was lowest under both parental genotypes and highest under both hybrids. Seed N uptake was lowest in the control plots (0 kg ha^−1^ fertilizer N) and increased with fertilizer application, though there were no differences among the urea-N rates (50, 100, and 150 kg ha^−1^; Figure 4). There was high variability in seed N uptake under the four genotypes and, like seed yield, parental genotype NAM-17 plateaued after the 50 kg ha^−1^ N fertilizer rate (Figure 4). Canola NUE (seed N uptake/urea-N fertilizer rate) and partial factor productivity (seed yield/urea-N fertilizer rate) varied among canola varieties and N fertilizer rates, and there was an interaction between the two factors (Table 2). Canola NUE and partial factor productivity were higher under both hybrids and lower under the parental genotypes, though the rankings of the parental genotypes did not align with their corresponding hybrids. Canola NUE and partial factor productivity were higher under the 50 kg ha^−1^ N fertilizer rate than the higher N fertilizer rates (Figure 5). Variability in NUE and partial factor productivity between genotypes decreased at the higher urea-N fertilizer rates (Figure 5).

### 2.3. Category A and B Variables’ Inter-Relationships

**Figure 6:**
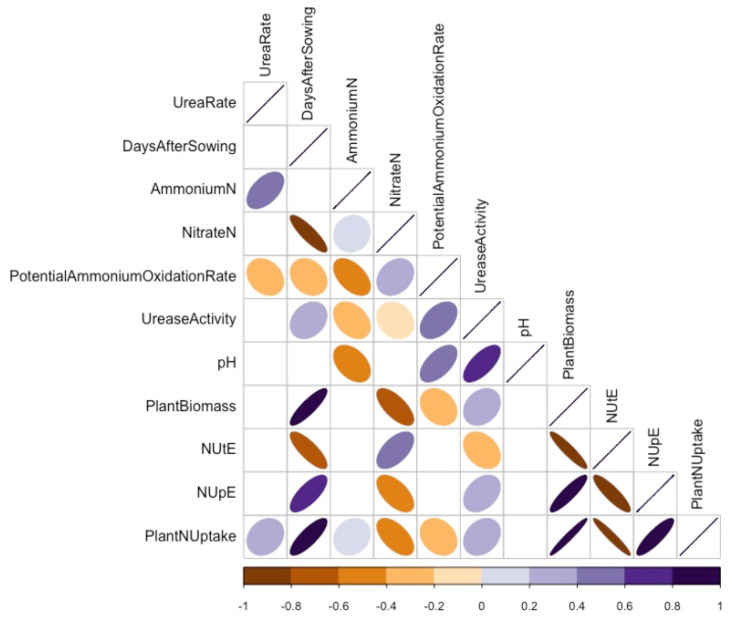
Spearman’s rank correlation coefficient (*p* < 0.05) of Category A variables showing correlation (r) among urea-N rates, days after sowing (DAS), plant biomass, nitrogen uptake efficiency (NUpE), nitrogen utilization efficiency (NUtE), ammonium-N, nitrate-N, ammonium oxidation, urease, plant N content, N uptake, and pH in varying canola varieties. Colours and shapes of ellipses indicate the strength of the correlations, with only significant correlations at a confidence level of 95% depicted. Positive and negative correlations are depicted by the respective direction of each ellipse, and positive correlations are depicted with the colour purple and negative correlations are depicted with the colour orange.

Spearman’s rank correlation coefficient was calculated based on the adjusted means of the variables of the Category A and B datasets. We conducted these correlations to determine the relationships between the above- and below-ground parameters that affected canola growth and seed production. For Category A variables (Figure 6), soil NO_3_^−^-N content negatively correlated with days after sowing (DAS), plant N uptake, NUpE, and plant biomass, and positively correlated with NUtE. Soil NH_4_^+^-N content negatively correlated with potential ammonium oxidation rates, urease activity, and soil pH and positively correlated with N fertilizer rate. Soil pH positively correlated with potential ammonium oxidation rates and urease activity and negatively correlated with N fertilizer rate. Also, above-ground plant biomass positively correlated with plant N uptake, NUpE, and DAS and negatively correlated with NUtE. Finally, NUpE was positively correlated with DAS urease activity and negatively correlated with NUtE, while NUtE correlated negatively with DAS. Nitrogen fertilizer rate negatively correlated with urease and potential ammonium oxidation rates.

**Figure 7:**
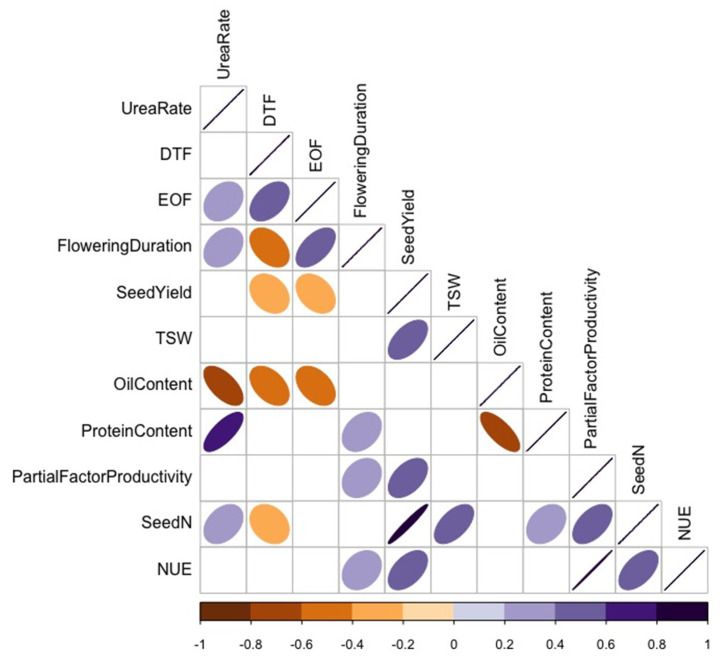
Spearman’s rank correlation coefficient (*p* < 0.05) of Category B variables showing correlation (r) among urea-N rate, % oil content, nitrogen use efficiency (NUE), thousand-seed weight (TSW), seed yield, seed N, start of flowering (DTF), % protein content, end of flowering (EOF), partial factor productivity, and duration of flowering in varying canola varieties. Colours and shapes of ellipses indicate the strength of the correlations, with only significant correlations at a confidence level of 95% depicted. Positive and negative correlations are depicted by the respective direction of each ellipse, and positive correlations are depicted with the colour purple and negative correlations are depicted with the colour orange.

For Category B variables (Figure 7), TSW positively correlated with seed N uptake and seed yield. Seed yield negatively correlated with the end of flowering and flowering duration and positively correlated with partial factor productivity, seed N uptake, and NUE. As expected, seed protein content negatively correlated with seed oil content, and canola NUE positively correlated with flowering duration, seed yield, and seed N uptake. The correlations between 1) NUE and flowering duration and 2) NUE and the end of flowering were only significant for parent varieties (*p* < 0.05). Fertilizer N rate was positively correlated with seed protein content and negatively correlated with seed oil content. Flowering duration positively correlated with the end of flowering.

Category A soil variables collected at the 5–6 leaf stage were compared to Category B yield and NUE harvest metrics. Canola NUE positively correlated with potential ammonium oxidation rates (r = 0.30, *p* = 0.0216), urease activity (r = 0.26, *p* = 0.0520, marginally significant), NUtE (r = 0.31, *p* = 0.0173), and NUpE (r = 0.45, *p* = 0.0005), and negatively correlated with NH_4_^+^-N concentrations (r = −0.52, *p* < 0.0001). Seed yield negatively correlated with soil pH (r = −0.46, *p* = 0.0004) and potential ammonium oxidation rates (r = −0.28, *p* = 0.0320), and positively correlated with soil NH_4_^+^-N concentrations (r = 0.39, *p* = 0.0024) and plant NUtE (r = 0.24, *p* = 0.0713, marginally significant). Similarly, seed N uptake negatively correlated with soil pH (r = −0.40, *p* = 0.0021) and potential ammonium oxidation (r = −0.31, *p* = 0.0200), and positively correlated with soil NH_4_^+^-N (r = 0.49, *p* < 0.0001) and NO_3_^−^-N concentrations (r = 0.26, *p* = 0.0535, marginally significant).

Category A soil variables collected at flowering were also compared to Category B yield and NUE harvest metrics. Canola NUE negatively correlated with soil NH_4_^+^-N (r = −0.38, *p* = 0.0027) and NO_3_^−^-N concentrations (r = −0.45, *p* = 0.0003), and positively correlated with plant NUtE (r = 0.45, *p* = 0.0003). Seed yield negatively correlated with soil pH (r = −0.42, *p* = 0.0008), potential ammonium oxidation rates (r = −0.28, *p* = 0.0328), and urease activity (r = −0.30, *p* = 0.0204), and positively correlated with plant NUtE (r = 0.42, *p* = 0.0008). Likewise, seed N uptake negatively correlated with soil pH (r = −0.40, *p* = 0.0018), potential ammonium oxidation rates (r = −0.29, *p* = 0.0240), and urease activity (r = −0.27, *p* = 0.0337), and positively correlated with plant NUtE (r = 0.32, *p* = 0.0124).

## 3. Discussion

Previous studies have found variation in NUE among crop varieties, but they have focused mainly on above-ground plant traits [17,18,43,44,45], without regard for soil N cycling processes. Therefore, we examined soil N cycling and NUE in canola hybrids compared to parental genotypes under varying N rates. We hypothesized that canola varieties with higher NUE (and NUpE and NUtE) would be associated with increased soil N cycling and passive plant traits like higher thousand-seed weight (TSW), earlier start to flowering, and longer flowering duration. Since passive plant traits can affect yield and NUE in hybrid canola [17], we hypothesized that these traits might also positively influence hybrid yield and NUE in our study. Understanding the soil N processes that affect canola NUE can further build on recent advancements in understanding NUE across different crops [46,47,48,49] and improvements in breeding for N-efficient crop varieties [30].

We explored the hypothesis that canola varieties with higher soil N cycling would have higher NUE. A recent study proposed that higher NUE in hybrid canola may be associated with increased below-ground interactions that result in better scavenging of native soil and fertilizer N [15]. Although NUE was higher under hybrids than parental varieties, we did not observe varietal differences in soil N cycling, but rather a strong phenological effect. We demonstrated that canola NUE correlated positively with soil urease activity and potential ammonium oxidation rates at the 5–6 leaf stage. Greater soil N cycling resulted in higher concentrations of plant-available N, though plant phenology constrained these results. Ammonium oxidation rates were higher earlier in the growing season, likely driven by urea-N fertilizer application, resulting in higher soil NO_3_^−^ concentrations for plant N uptake. As plants developed, soil NO_3_^−^-N concentrations declined, likely due to increased canola N demands [50]. Plant N uptake negatively correlated with soil NO_3_^−^-N concentrations, supporting this idea [39]. Therefore, the production of soil mineral N through soil N cycling likely resulted in high canola NUE, with greater soil N availability resulting in higher soil N acquisition. Our sampling depth was limited to the soil surface; expanding sampling depth, possibly to 40 cm, may better explain genotypic differences in soil N availability and N cycling since up to 85% of the canola root mass is located in the 0–40 cm soil depth [51]).

Soil NO_3_^−^-N concentrations declined as plants were in their reproductive phase and N demands increased, but soil NH_4_^+^-N increased and was unexpectedly higher than NO_3_^−^-N at flowering. These dynamics may be due to canola’s preference for soil NO_3_^−^ over NH_4_^+^ [39] and changing dynamics in soil N cycling. For instance, NH_4_^+^ produced from higher mineralization (increase in urease activity) at flowering may not have been rapidly nitrified (decline in potential ammonium oxidation rates). Indeed, N mineralization may have been stimulated at flowering by root exudates when root exudation would be expectedly high because of greater root biomass [52,53,54,55]. Previous work has shown an increase in canola root biomass between late flowering and late pod filling [51]. Further, in a previous study, soil NO_3_^−^ increased from the seedling to late-flowering stages, decreased between late flowering and late pod filling, and increased after that [50]. With the limited phenological stages in this study, it is likely that if we expanded our scope to include the seed ripening stage, we would have observed an increase in NO_3_^−^-N as the plants matured.

Canola N uptake increased with increasing urea-N fertilizer rates, as was expected [48,56], but a fertilizer N rate and variety interaction meant that varieties with similar N uptake at the lower fertilizer N rates did not have similar N uptake at the higher N rates. Parent and hybrid pairs had similar N accumulation at low N rates, while hybrids were more similar at the 100 kg ha^−1^ and 150 kg ha^−1^ N rates. We posit that hybrid and parent pairs had similar N uptake at lower urea-N fertilizer rates because of genotypic traits between the pairs and because hybrids were not receiving optimal N. At the 100 kg ha^−1^ and 150 kg ha^−1^ N rates, N uptake increased under both parents and hybrids. However, hybrids outperformed the parental varieties at these higher N rates, acquiring more N. Some studies revealed that hybrid canola responded to higher N availability over parental varieties because the N requirements for hybrids were higher than parental varieties, needing higher rates of fertilizer N [57,58]. However, NUpE did not follow the same patterns (though only marginally significant) and was higher at the 0 kg ha^−1^ N rate and lower at the 150 kg ha^−1^ N rate. There likely was an oversupply of N at the 150 kg ha^−1^ N rate. Canola NUtE decreased with increasing N rates, suggesting low N recovery and remobilization when fertilizer N was high. Our results also suggest that because NUpE and NUtE (significant predictors of canola NUE [17]) were lower at the higher N rates [56], there is a need to proportion fertilizer N rates to improve NUE. Indeed, canola NUE decreased with increasing urea-N fertilizer rates, possibly because of the limited ability of these varieties to transport plant N to seeds during N remobilization.

We predicted that soil N cycling rates would also respond positively to urea-N fertilizer rates, but neither urease activity nor potential ammonium oxidation rates were affected by urea-N fertilizer rates. For urease, this supports similar results from one meta-analysis [59]. However, in two more comprehensive meta-analyses, each analyzing larger datasets, it was determined that urease activity increased with increasing N rates in agricultural soils [40,60]. Similarly, a recent meta-analysis [61] indicated that potential ammonium oxidation rates increased with increasing fertilizer rates, contradicting our findings. It is possible that soil N cycling did not increase with increased urea-N fertilizer application because N was not deficient (though marginal based on pre-seeding soil tests) in our soil, providing native soil N for mineralization and nitrification.

Canola N uptake was more similar between corresponding parent and hybrid varieties, especially when plants were young. For example, at the 5–6 leaf stage, parental genotype NAM-0 and corresponding hybrid H151857 had higher NUpE than parental genotype NAM-17 and its corresponding hybrid, H151816. However, all varieties had similar NUpE rates by flowering, but these were more similar between related parent and hybrid varieties, suggesting an inherited trait for NUpE. Further, soil NO_3_^−^-N differences were not distinguishable between parent vs. hybrid varieties, but rather corresponding hybrid–parent pairs. For instance, at the 5–6 leaf stage, lower soil NO_3_^−^-N concentrations under parent genotype NAM-0 and corresponding hybrid H151857 indicated that plants absorbed much of the soil NO_3_^−^-N (or microorganisms immobilized soil NO_3_^−^-N) more rapidly than the NAM-17 and its corresponding hybrid. Though NAM-17 and its corresponding hybrid, H151816, had lower NUpE than NAM-0 and its corresponding hybrid, H151857, hybrid H151816 had higher NUtE, indicating an enhanced ability to remobilize plant N, even with reduced soil N absorption. At flowering, there were varietal-specific traits that potentially resulted in higher NUE. For example, hybrids surpassed the parental varieties in NUtE, ultimately leading to higher seed N uptake, yield, and NUE. Indeed, parent genotype NAM-17 was the most inefficient at N utilization at flowering and had the lowest yield and NUE. Further, NAM-17 seed yield stagnated after 50 kg ha^−1^ N because it had the lowest yield potential, whereas the other genotypes were more responsive to increasing urea-N fertilizer rates, at least up to 100 kg N ha^−1^. At 150 kg N ha^−1^, yields declined, suggesting that excessive N [62,63] likely caused lodging [64,65,66].

Plant N remobilization is important in canola NUE because when canola shifts from the vegetative to the generative phase, leaves senesce and are aborted before seed maturity [18,67]. Therefore, for increased NUE, N must be remobilized from leaves to stems, then to siliques, and finally to seeds under cases of complete remobilization [18,67]. Both hybrids had high NUtE at the 5–6 leaf stage and flowering. Therefore, higher NUtE might play a more critical role in high NUE in canola varieties than NUpE. Differences in maturity time may also explain varietal differences in NUE. Varieties that shift earlier from the vegetative to the generative developmental stage can have higher NUE because of the extended time for N remobilization and the longer seed developmental period [18]. Parent genotype NAM-17 took the longest to transition from the vegetative to the generative stage, was inefficient in N utilization, and had the lowest NUE. However, our results demonstrated that longer flowering duration did not necessarily lead to higher NUE. While flowering duration correlated positively with NUE, only hybrid variety H151857 had higher NUE and prolonged flowering duration. Parent genotype NAM-0 had a long flowering duration but did not have a high NUE. While an early switch from the vegetative to the generative stage can result in higher NUE [18], this increase may be limited to phenological stages after flowering, such as seed pod filling [67,68], and seems to be more varietal driven, though further research is needed.

We also explored the hypothesis that canola varieties with higher passive plant yield components would have higher NUE. Passive traits, such as TSW, may influence crop NUE—Stahl et al. determined that higher hybrid canola NUE related to increased seed TSW [17]. We also found that hybrids had higher seed TSW, but this did not correlate with NUE. We determined that NAM-17 and its corresponding hybrid, H151816, had a shorter flowering duration and later onset of flowering overall; this meant a prolonged switch from vegetative to generative growth, allowing for an extended period for N absorption and remobilization. At least for H151816, these passive traits could have allowed for higher NUE, while the hybrid variety H151857 could have had higher NUE because of its ability for high soil nitrate absorption earlier in the growing season.

## 4. Materials and Methods

### 4.1. Site Characteristics

The field experiment was conducted on a dark brown chernozem over one growing season in 2018 at the Agriculture and Agri-Food Canada Saskatoon Research Centre Farm in Saskatoon, SK, Canada (52°9′2.52″ N, 106°32′37.32″ W). Wheat was grown in this field before the present study. Table S1 reports the average daily spring and summer temperature and rainfall for the growing season [69], and Table S2 indicates pre-seeding soil properties, sowing date, and maturity date. Soil inorganic N prior to seeding was 26.9 kg ha^−1^ in the 0–15 cm soil profile and 56.0 kg ha^−1^ in the 15–60 cm soil profile, representing marginal N in relation to fertility recommendations for optimal yield.

### 4.2. Field Management and Experimental Design

The experimental design was a randomized split-plot design with four replications. Each main plot was 1.2 m wide and 5.94 m long and contained four rows spaced 0.3 m apart. The spacing between each replicate block—gaps between plots planted with barley within the blocks—was 0.6 m. Four urea-N fertilizer rates (0, 50, 100, and 150 kg ha^−1^) were randomly assigned to the main plots and four canola varieties (described below) to the sub-plots. The treatments were replicated four times to give a total of 64 experimental units. Seeds from all varieties were sown in rows at a 1.27 cm depth in May 2018, with a seeding rate of 1027 g/plot; seeds were pretreated with insecticide and fungicide (Helix^®^ Vibrance^®^; Syngenta Canada Inc., Guelph, ON, Canada) before planting. Nitrogen was applied as urea (46% N) and was mid-row banded at seeding at rates of 50, 100, and 150 kg N ha^−1^. Control plots that did not receive urea (0 kg N ha^−1^) were included. Fertilizers for sulfur and phosphorus were added pre-seeding as 23.3 kg ha^−1^ ammonium sulfate and 39.8 kg ha^−1^ mono-ammonium phosphate, while potassium (K) fertilizer was not required due to naturally high K fertility in these soils (Table S2). Edge^®^ granular herbicide (Gowan Company, Yuma, AZ, USA) was applied to the field in spring at 20.5 kg ha^−1^.

### 4.3. Canola Varieties

This study was comprised of four spring growth *B. napus* varieties: two parental genotypes and two experimental hybrids. Parental genotypes included NAM-0 and NAM-17, both from the Agriculture and Agri-Food Canada canola breeding program. The two genotypes were breeding lines selected for production in western Canada but had genetically different germplasms. The experimental hybrid combinations for this study included H151816 (where parental genotype NAM-17 was the male crossed with a female tester) and H151857 (where parental genotype NAM-0 was crossed with the same female tester).

### 4.4. Plant and Soil Sampling and Biomass Determination

Canola growth and development were recorded based on phenological growth stage [68]—above-ground plant samples were collected at the 5–6 leaf stage, flowering, and harvest maturity. The 5–6 leaf stage encompassed varieties at the 5-leaf, 6-leaf, and 5–6 leaf transition stage because of the varying emergence and growth rates among the different varieties. At the 5–6 leaf stage and flowering, plant roots and root-associated soils were collected using a soil corer (10 cm in length × 5 cm in diameter). We focused on the surface–10 cm depth because of higher soil microbial activity in this soil layer [70] and because soil nutrients and water are typically concentrated in this layer [51]. Canola roots were removed from the soil sample, and the soils were sieved (2 mm) and stored at −20 °C for nutrient and biochemical analyses. The above-ground plant material was cut from the roots and oven-dried at 60 °C for 72 h, then weighed. At harvest maturity, above-ground plant material was collected, and the number of plants was counted on an area basis (0.5 m × 0.5 m). Above-ground plant biomass was determined by multiplying individual plant dry biomass by the number of plants per 0.5 m × 0.5 m area. The start of flowering was determined when >50% of the plot had a minimum of one flower, the end of flowering was determined when >90% of the plants had no flowers remaining, and flowering duration was calculated as the number of days from the start of flowering to the end of flowering. Physiological maturity was determined when 50% of the seed color changed halfway up the main raceme, and harvest maturity represented the date when plants were harvested through direct cut. At maturity, the seed was mechanically harvested, and seed yield was determined by dividing the seed weight collected within a plot by the plot size per unit area. The thousand-seed weight (TSW) was determined based on the counting and weighing of 1000 seeds twice, from a representative 100 g aliquot from each sample.

### 4.5. Canola N Uptake and NUE Metrics

Total N concentration was determined for finely ground above-ground plant tissue samples and whole seed samples following the Dumas combustion protocol [71,72] using a TruMac analyzer (LECO Corporation, MI, USA). Nitrogen uptake was calculated by multiplying the amount of N at the individual level by the ratio of plant dry biomass per hectare over the individual plant dry biomass [17]. Seed oil and protein content were determined by near-infrared reflectance (NIR) [73]. A Foss NIRSystems Model 6500 analyzer calibrated with appropriate oilseed samples was used. The NIRSystems Model 6500 was calibrated with oilseed samples extracted with hexane [74] for oil content, and the NIRSystems Model 6500 was calibrated with oilseed samples whose protein contents had been determined prior [75] for protein content. Results were reported as a percentage on a whole seed dry matter (zero moisture) basis, and the NIR calibrations used in this study were certified through the Canadian Grain Commission’s WCC/RRC Laboratory Proficiency Program using known check samples.

Canola NUE metrics were calculated as suggested by Gan et al. [15] and Stahl et al. [17]. Canola NUpE was calculated by dividing the total above-ground plant N uptake by urea-N supplied, NUpE = plant N/(Nt + Nf). Specifically, Nt represents the N derived from the soil determined from the N uptake in above-ground plant biomass in control plots where no urea-N fertilizer was applied and soil was the sole N source, while Nf represents the amount of urea-N fertilizer applied [15]. Canola NUpE was determined at the 5–6 leaf stage and flowering because canola drops most of its leaves after flowering, and leaves represent a large N sink [18]. Canola NUtE was calculated as the ratio of seed yield at harvest maturity to N uptake in the plants at the 5–6 leaf stage and flowering (seed yield/plant N uptake) to account for the net effects of subcellular N recycling in the plants before leaves are lost [18]. Canola NUE was calculated by dividing seed N uptake by urea-N fertilizer [76,77], and partial factor productivity (PFP) was calculated by dividing seed yield by urea-N fertilizer [78].

### 4.6. Soil Analyses

Soil NO_3_^−^-N and NH_4_^+^-N were extracted using 2 M KCl, where field-fresh soil was weighed (5 g) and 50 mL of 2 M KCl was added to each sample [79]. Samples were shaken at 160 rpm for 30 min, then filtered through Whatman 42 filter paper. Filtered samples were analyzed using a Technicon Autoanalyzer (Technicon Industrial Systems, Tarrytown, NY, USA). Urease activity was determined for each sample [80]. Briefly, 5 g of each soil sample was weighed in triplicate and 2.5 mL of 720 mM urea substrate was added to two of the triplicates, leaving the third triplicate as a control. Next, 20 mL of 0.1 M borate buffer (pH = 10) was added to all samples, including the control, and samples were incubated for 2 h at 37 °C. After incubation, 2.5 mL of the substrate was added to the control solution, and the release of NH_4_^+^-N was extracted using 30 mL of a 2 M KCl–0.01 M HCl solution. The samples were shaken on a rotary shaker at 150 rpm for 30 min, after which the soil suspension was filtered. Samples were quantified along an NH_4_Cl calibration curve at 660 nm using a spectrophotometer (Thermo Scientific™ Evolution 60S) to determine the NH_4_^+^-N released. Potential ammonium oxidation rates, a proxy for ammonia monooxygenase activity, were determined [80]. Triplicate subsamples of 5 g of soil were weighed, and 20 mL of 1 mM ammonium sulfate solution and 0.1 mL of 1.5 M sodium chlorate solution were added to all triplicates. Two of the triplicates were incubated at 21 °C on a rotary shaker (150 rpm), and the third triplicate sample, serving as the control, was incubated at −20 °C; all samples were incubated for 5 h. After incubation, the control was thawed at room temperature, and 5 mL of 2 M KCl was added to the two triplicates, which were immediately filtered. The same was done for the control sample once it was thawed. Samples were quantified along a sodium nitrite (NaNO_2_) calibration curve at 520 nm using a spectrophotometer (Thermo Scientific™ Evolution 60S) to determine the nitrite released. Soil pH was determined by suspending air-dried soil samples in a 1:2 weight: volume ratio of 0.01 M CaCl_2_ solution [81].

### 4.7. Data Analysis

Our data were divided into two categories, Category A and Category B. Category A included soil and plant data collected from plot composite soil and plant samples at the 5–6 leaf stage and flowering, while Category B included plant data collected at harvest maturity on a per plot level, as well as days to flowering and flowering duration. The Category A data included the soil inorganic N and soil N cycling rates, plant biomass and N uptake, NUtE, and NUpE, while the Category B data included TSW, seed yield, seed N uptake, seed oil concentration, protein concentration, and NUE and PFP (partial factor productivity). Category A data were analyzed using mixed-effects analysis of variance with canola variety, N fertilizer rate, and phenological stage as fixed factors and replicate block and N fertilizer rate as the random factors. Category B data were also analyzed using mixed-effects analysis of variance with variety and N fertilizer rate as fixed factors and replicate block and N fertilizer rate as the random factors. For Category A and B models, variety was nested within N rate to account for the split-plot design. Canola varieties were nested within N rates because N rates were randomly assigned to each plot, then for each plot, canola varieties were randomly assigned to the split portions, creating different levels of randomization applied to each subject [82]. Data were tested for normality using the Shapiro–Wilk test—normality was assumed if *p*-values were greater than 0.05. Tukey HSD post hoc tests were used to determine significant differences between treatments.

All statistical analyses were conducted in R [83] using the packages ggpubr [84], nlme [85], lsmeans [86], and multcomp [87] and data visualization was conducted using the packages plyr [88], reshape2 [89], lattice [90], gridExtra [91], and ggplot2 [92]. Spearman’s rank correlation coefficient was conducted for all canola varieties with *n* = 128 (Category A variables, among phenological stages, block, and N rate) and *n* = 64 (Category B variables, among block and N rate), using the package Hmisc [93] for statistics and corrplot [94] for plotting.

## 5. Conclusions

This study revealed complex N dynamics in below-ground soil N processing and canola NUE. The objective of this study was to evaluate how canola varieties (hybrids vs. parent) under varying fertilizer urea-N rates affect (1) soil N cycling processes, (2) soil N concentration, and (3) plant NUE (including NUpE and NUtE); and how this varies over the growing season. We observed significant varietal differences in soil NO_3_^−^-N concentrations associated with higher plant NUpE at flowering when plant demand for N was higher. Hybrids had significantly higher seed yield and seed N allocation. Varietal differences in seed N uptake, seed yield, NUtE, and NUE related to hybrid canola efficiently using soil N and allocating N to the seeds. Hybrids had the highest NUE compared to the parental genotypes, potentially owing to their ability to have high NUpE (hybrid H151816 at flowering and H151857 at the 5–6 leaf stage) and high NUtE at flowering, a known bottleneck to canola NUE. Increasing urea-N fertilizer rates did not result in higher NUE, possibly because of saturated N uptake ability or N remobilization (NUpE and NUtE were highest at the lower N rates). The efficiency of hybrids to remobilize N for seed production allowed these varieties to maintain productivity even at the lower N rates. Beyond varietal differences, we demonstrated that canola NUE increased with decreasing soil mineral N—linked to increased N uptake. We also demonstrated critical relationships between soil N cycling and canola NUE, where soil N cycling positively correlated with NUE. Future studies should expand our knowledge of below-ground dynamics, focusing on microbial N cycling and soil N status, root exudate, and root system architecture, extending our measurements to greater soil depths. Future studies should also expand our knowledge of passive traits that affect NUE, especially the onset and duration of flowering. Varieties with extended vegetative periods should be selected because they will likely have higher N absorption and N remobilization with a prolonged vegetative stage.

## Figures and Tables

**Table 1:** ANOVA of Category A variables under four varying urea-N fertilizer rates. V: canola varieties; NR: urea-N fertilizer rates; PS: phenological stage; NUtE: nitrogen utilization efficiency; NUpE: nitrogen uptake efficiency.

Factors	Urease Rate (μg NH_4_^+^ g^−1^ 2 h^−1^)	Potential Ammonium Oxidation Rate (μg NO_2_^−^-N g^−1^ 5 h^−1^)	Soil NO_3_^−^-N (mg kg^−1^)	Soil NH_4_^+^-N (mg kg^−1^)	Soil pH	Plant Biomass (kg ha^−1^)	N Uptake (kg ha^−1^)	NUtE	NUpE
V	0.4652	0.6585	0.3542	0.6169	0.9723	0.5262	0.6483	**0.0010**	0.9405
NR	0.7557	0.5350	**0.0010**	**0.0002**	0.6209	0.2569	**0.0260**	*0.0944*	*0.0703*
PS	**0.0001**	**0.0003**	**<0.0001**	0.8743	**0.0069**	**<0.0001**	**<0.0001**	**<0.0001**	**<0.0001**
V × NR	0.8916	0.9317	0.5148	0.4735	0.3033	*0.0777*	**0.0442**	0.1118	*0.0549*
V × PS	0.3135	0.9281	**0.0055**	0.1268	0.9152	**0.0001**	**0.0001**	**0.0003**	**0.0029**
NR × PS	0.1238	0.1623	0.2954	**0.0072**	0.9829	0.7930	0.1824	0.2440	**0.0107**
V × NR × PS	0.8061	0.2085	0.2273	0.9610	0.9677	0.1556	0.1868	0.2424	0.1588

Significant at the 0.05 probability level is represented in bold font. Significance at the 0.1 probability level is represented in italic font.

**Table 2:** ANOVA of Category B variables under four varying urea-N fertilizer rates. V: canola varieties; NR: urea-N fertilizer rates; TSW: thousand-seed weight; NUE: nitrogen use efficiency.

Factors	Start of Flowering (Julian Days)	End of Flowering (Julian Days)	Flowering Duration (No. of Days)	Yield (kg ha^−1^)	Seed N (kg ha^−1^ N)	TSW (g)	Seed Oil (%)	Seed Protein (%)	Partial-Factor Productivity	NUE
Treatments										
V	**<0.0001**	**<0.0001**	**<0.0001**	**<0.0001**	**<0.0001**	**<0.0001**	**0.0001**	0.3820	**<0.0001**	**<0.0001**
NR	0.3128	**0.0137**	**0.0025**	**0.0149**	**0.0006**	0.4138	**0.0040**	**0.0015**	**<0.0001**	**<0.0001**
Interaction										
V × NR	**0.0350**	0.2819	0.4411	0.3764	0.3163	0.6557	0.7850	0.8069	0.6689	0.8216

Significant at the 0.05 probability level is represented in bold font.

## Data Availability

The data presented in this study are openly available in FigShare: “Canola nitrogen use efficiency and soil nitrogen cycling in response to varying nitrogen fertilizer rates across hybrid canola varieties and their parents”. Direct URL to data: https://doi.org/10.6084/m9.figshare.16654804.v4 (accessed on 21 September 2021) [95].

## Supplementary Materials

The following are available online at https://www.mdpi.com/article/10.3390/plants10112364/s1, Figure S1: Above-ground plant biomass over four diverse canola varieties and four varying N treatment rates at two phenological growth stages (5–6 leaf stage and flowering, *n* = 4). Figure S2: Canola N uptake over four diverse canola varieties and four varying N treatment rates at two phenological growth stages (5–6 leaf stage and flowering, *n* = 4). Table S1: Meteorological condition during the canola growing period for the 2018 season. Meteorological data obtained from Environment and Climate Change Canada. Table S2: Physio-chemical properties of study site prior to seeding in 2018.
