# Supplementary material for: Nitrogen Use Efficiency in Parent vs. Hybrid Canola under Varying Nitrogen Availabilities
Source: Plants (Basel). 2021 Nov 2;10(11):2364. doi: 10.3390/plants10112364 (PMC8623409; doi:10.3390/plants10112364)
Supplement: Supplementary file 1 [file plants-10-02364-s001.zip › plants-1424529-supplementary.pdf]

Supplementary Material

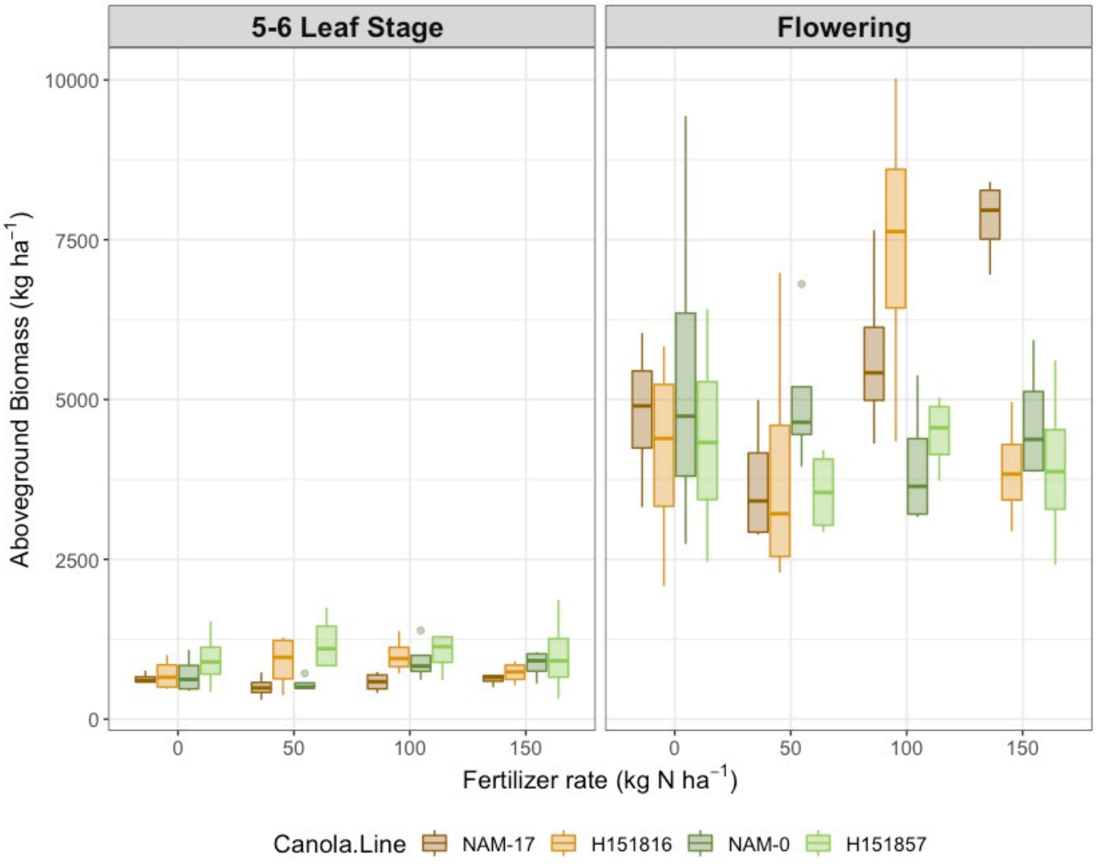

Figure S1| Aboveground plant biomass over four diverse canola varieties and four varying N treatment rates at two phenological growth stages (5-6 leaf stage and flowering, n = 4).

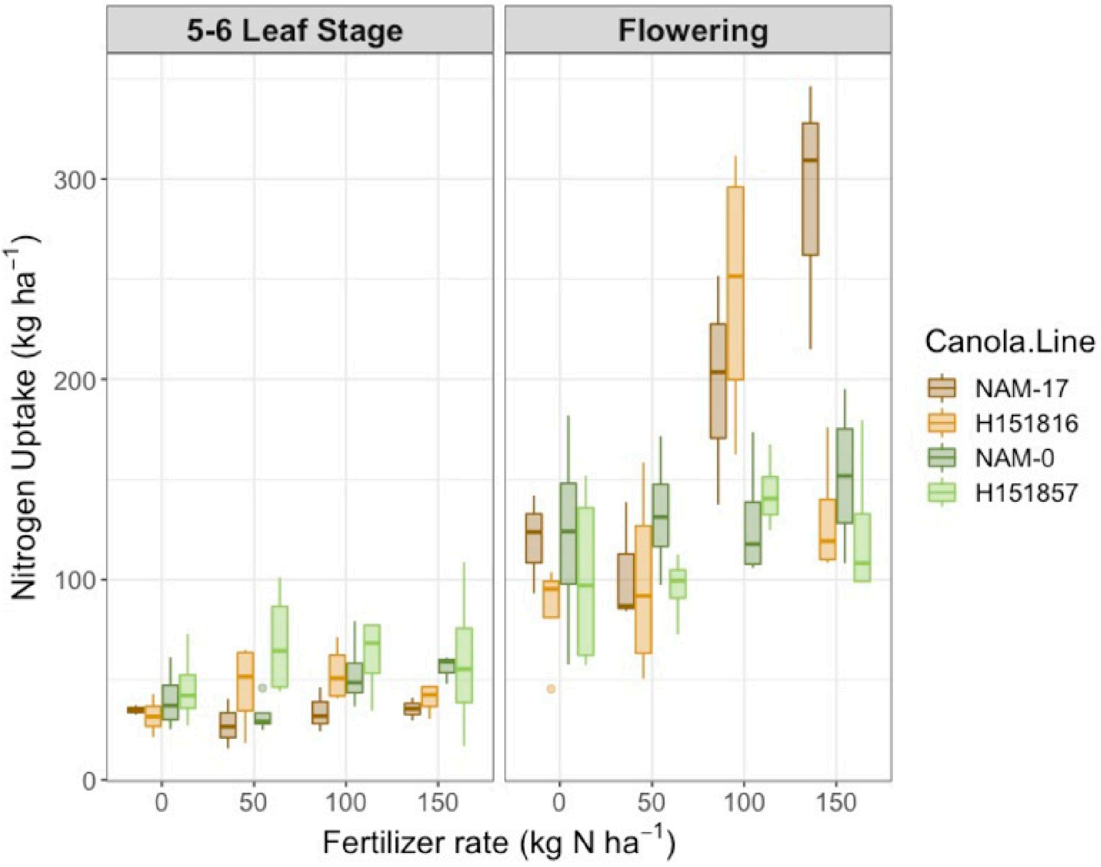

Figure S2| Canola N uptake over four diverse canola varieties and four varying N treatment rates at two phenological growth stages (5-6 leaf stage and flowering, n = 4).

Table S1| Meteorological condition during the canola growing for 2018 season. Meteorological data obtained from Environment and Climate Change Canada https://climate.weather.gc.ca/climate_data.

**Growing months in 2018**	**Broad Sampling stages**	**Mean daily precipitation (mm)**	**Mean daily temperature (°C)**
May	Seeding	35.0	14.3
June	5-6 leaf stage	19.9	17.3
July	Flowering	31.1	18.7
August	Physiological maturity	17.2	17.1
September	Harvest maturity	37.1	7.4

Table S2| Physio-chemical properties of study site prior to seeding in 2018.

**Variables**	**Llewellyn Farm 2018**
	**Soil Properties (0-15 cm depth)**
Soil pH	7.3
Organic matter (%)	5.5
Electrical conductivity (dS/m)	0.6
Available N (ppm)[1]	12.0**
Available P (ppm)	23.0**
Available K (ppm)	780.0⇑
Available S (ppm)	8.0•
Available Ca (ppm)	5900.0•
Available Mg (ppm)	960.0•
Available Na (ppm)	27.0⇓
	**Seeding Factors**
Preceding crop	Wheat
Seeding date	Julian day 147
Emergence rate	30-100% emergence; 51% average emergence
Days to harvest maturity	77-85 day range after seeding; 80 days average

⇓ Deficient, ** Marginal, • Optimum, ⇑ Excess in relation to fertility recommendations for optimal yield. [1]Available N was examined at two depths pre-seeding, with 12 ppm (26.9 kg ha^{-1}) available N at the 0-15 cm depth and 8 ppm (56.0 kg ha^{-1}) at the 15-60 cm depth.
